# Comparative associations and incremental predictive value of cardiometabolic and inflammatory indices for short- to mid-term cardiovascular disease risk in two Chinese cohorts

**DOI:** 10.3389/fcvm.2026.1896146

**Published:** 2026-08-06

**Authors:** Tumalisi Julaiti, Qinghua Chen, Junxi Lu, Xian Chen, Xuhong Hou, Jie Zhang, Jiemin Pan

**Affiliations:** 1 Department of Endocrinology and Metabolism, Shanghai Key Laboratory of Diabetes Mellitus, Shanghai Diabetes Institute, Shanghai Sixth People's Hospital Affiliated to Shanghai Jiao Tong University School of Medicine, Shanghai, China; 2 SenseTime, Shenzhen, China; 3 Department of Information Technology, Haikou Orthopedic and Diabetes Hospital, Haikou Orthopedic and Diabetes Hospital of Shanghai Sixth People's Hospital, Haikou, China

**Keywords:** cardiometabolic risk, cardiovascular disease, Chinese visceral adiposity index, risk prediction, visceral adiposity

## Abstract

**Background:**

Composite cardiometabolic and inflammatory indices can be calculated from routine anthropometric and laboratory measures, but their value for cardiovascular disease (CVD) risk assessment in Chinese populations remain unclear.

**Methods:**

We analyzed 7,170 participants free of CVD at baseline from the China Health and Retirement Longitudinal Study (CHARLS) and the Shanghai Diabetes Study (SHDS). Mean follow-up was approximately 41 months in both cohorts. The evaluated indices included body mass index (BMI), Chinese visceral adiposity index (CVAI), lipid accumulation product (LAP), triglyceride-glucose index (TyG), atherogenic index of plasma (AIP), high-sensitivity C-reactive protein (hs-CRP), hs-CRP to high-density lipoprotein cholesterol ratio (hs-CRP/HDL-C), and hs-CRP-glucose index (CTI). Cox proportional hazards models were used to examine associations with incident CVD, with cohort interactions tested to assess cross-cohort consistency. Dose-response and nonlinearity were evaluated using quartile-based analyses and restricted cubic splines. Predictive performance and the added value of each index beyond a common clinical base model were evaluated in terms of clinical utility.

**Results:**

During follow-up, 343 CVD events occurred in CHARLS and 148 occurred in SHDS. CVAI was the only index consistently associated with incident CVD across both cohorts and adjustment models. In the fully adjusted model, the HR per SD increase in CVAI was 1.221 (95% CI: 1.070–1.392, *P* = 0.003) in CHARLS, and 1.330 (95% CI: 1.067–1.656, *P* = 0.011) in SHDS. Other indices showed cohort-specific patterns: CTI, hs-CRP, and hs-CRP/HDL-C remained associated with CVD in CHARLS, whereas LAP remained associated across models in SHDS. Quartile-based and spline analyses generally supported approximately linear dose-response patterns. When added to the clinical base model, individual indices produced only modest changes in discrimination, and decision curve net benefit.

**Conclusions:**

In two Chinese cohorts, CVAI showed the most reproducible association with short- to mid-term CVD risk, whereas other indices provided more cohort-specific signals. These indices may help describe cardiometabolic risk burden, but their incremental predictive value beyond conventional clinical factors was limited. Further studies with longer follow-up, verified outcomes, and more diverse populations are needed.

## 1 Introduction

Cardiovascular diseases (CVD) are the leading cause of death among non-communicable diseases worldwide and significantly reduce patients' quality of life (1). Early prediction of CVD plays a crucial role in enabling timely interventions and personalized treatment strategies. Atherosclerosis is the primary pathological basis of CVD (2, 3) and is regarded as a chronic inflammatory condition affecting medium- to large-sized arteries. Its development involves lipid deposition in the arterial intima (4), endothelial dysfunction, and infiltration of inflammatory cells (5). These pathological processes highlight the central roles of lipid dysregulation and chronic inflammation in the development of atherosclerosis and CVD. Therefore, in clinical practice, lipid profiling is considered a fundamental component for assessing CVD risk, while high-sensitivity C-reactive protein (hs-CRP) can serve as an adjunctive marker for risk stratification in certain populations (6–8). Insulin resistance (IR), a key metabolic driver of atherosclerosis, accelerates the initiation and progression of atherosclerotic lesions by disrupting lipid metabolism, promoting atherogenic dyslipidemia, chronic inflammation, and impairing endothelial function (9–11).

Conventional CVD risk assessment usually includes age, gender, blood pressure, lipid levels, smoking, diabetes, and hypertension. These variables are useful, but they may not fully describe visceral adiposity, insulin resistance, atherogenic dyslipidemia, or low-grade inflammation, which may be particularly relevant in middle-aged and older Chinese adults. Composite indices derived from routinely available blood tests and anthropometric measures may help summarize these related risk domains. For example, the triglyceride-glucose (TyG) index, derived from fasting plasma glucose (FPG) and triglyceride (TG) levels, is commonly used as a simple marker for insulin resistance and has shown stronger predictive power for cardiovascular risk in some studies (12–14). The Chinese visceral adiposity index (CVAI) (15) and the lipid accumulation product (LAP) (16) combine anthropometric and lipid measures to reflect visceral adiposity. The atherogenic index of plasma (AIP), defined as log_10_ (TG/HDL-C), reflects atherogenic dyslipidemia (17). More recently, the hs-CRP-TyG index (CTI) further integrates hs-CRP and TyG to present both low-grade inflammation and insulin resistance (18).

We hypothesized that composite indices integrating different cardiometabolic or inflammatory features would be associated with CVD and might add information beyond conventional clinical predictors. To evaluate this, we analyzed two independent Chinese cohorts recruited in different periods and settings. Studying both cohorts allowed us to assess whether the findings were consistent across populations with different baseline characteristics and follow-up conditions. Although several of these indices have been studied in relation to CVD risk, few studies have evaluated them in the same analysis across independent Chinese cohorts.

In this study, we aim to evaluate the associations and incremental predictive value of eight such measures for short- to mid-term incident CVD. We also assessed whether each index improved prediction beyond a common clinical base model.

## 2 Materials and methods

### 2.1 Study population

This study utilized data from two cohort studies of the Chinese population: the China Health and Retirement Longitudinal Study (CHARLS) and the Shanghai Diabetes Study (SHDS). CHARLS is an ongoing longitudinal study conducted by Peking University, launched in 2011 with a follow-up survey every two years (19). We included participants with Wave 1 (2011) as baseline and follow-up data from Wave 2 (2013) or Wave 3 (2015). CHARLS data are publicly available; detailed methodology and documentation can be accessed on the official website (https://charls.charlsdata.com/). The SHDS was a cross-sectional epidemiological survey of diabetes mellitus and impaired glucose regulation in Shanghai, China, from 1998 to 2001, which served as the baseline for the present study (20–22). Participants were followed up from December 2003 to November 2004, during which incident CVD events were reviewed and health examinations repeated.

**Figure 1:**
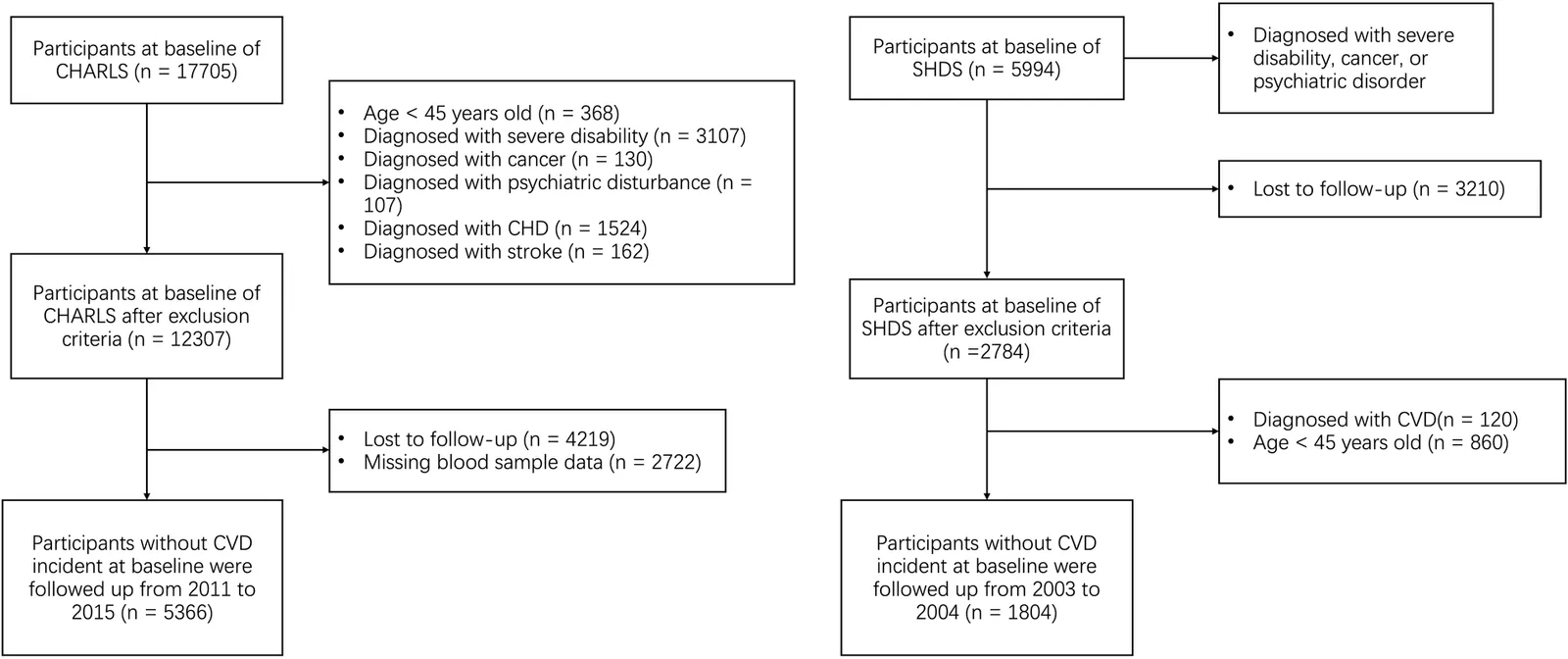
The flowchart of the study.

The participant selection process is summarized in Figure 1. At baseline, we excluded individuals from both populations who self-reported having pre-existing coronary heart disease (CHD), stroke, cancer, severe disability, psychiatric conditions, or who were under 45 years of age. Identical exclusion criteria were applied to both cohorts. In SHDS, variables with >20% missingness were excluded (the six key biomarkers had <1% missing in this cohort); in CHARLS, participants with missing values in any biomarker component for the exposure indices were removed. All participants provided informed consent after receiving detailed written information about the studies. The CHARLS study was approved by the Biomedical Ethics Review Committee of Peking University, Beijing, China (IRB00001052–11015). The SHDS study was approved by the Ethics Committee of Shanghai Jiao Tong University Affiliated Sixth People's Hospital.

### 2.2 Variables

#### 2.2.1 Covariates

In Both studies, trained healthcare staff collected data through in-person interviews, physical examinations, and structured questionnaires. The harmonized variables included: (1) sociodemographic factors: age, gender, education level, smoking habit, and drinking habit; (2) chronic disease status: defined by self-reported doctor diagnosis for hypertension and diabetes mellitus. In CHARLS, baseline hypertension and diabetes mellitus status were further refined using questionnaire information on the use of corresponding antihypertensive or antidiabetic medications; such medication-based refinement was not applied in SHDS because comparable medication-indication information was unavailable. (3) biochemical markers: fasting blood samples (fasting time ≥8 h) tested for hs-CRP, TG, total cholesterol (TC), high-density lipoprotein cholesterol (HDL-C), low-density lipoprotein cholesterol (LDL-C), and FPG; and (4) anthropometric measures: height, weight, waist circumference (WC), and blood pressure (measured two to three times). Detailed data collection procedures have been described in previous publications for both cohorts (19–22).

#### 2.2.2 Endpoint

Endpoint Events were identified from questionnaire-based information in both cohorts. The primary endpoint was incident CVD, defined as a composite endpoint of either incident CHD or incident stroke, whichever occurred first. CVD event time was defined as the interval from the baseline interview to the follow-up interview at which the event was first reported, or to the last follow-up date for censored participants who remained event-free. In CHARLS, CHD or stroke was defined by a “yes” response to the question “Has a doctor ever told you that you have had a heart attack, coronary heart disease, angina, congestive heart failure, or other heart problems?” (for CHD) or “Has a doctor ever told you that you have had a stroke?” (for stroke). In SHDS, CHD was defined as self-reported doctor-diagnosed myocardial infarction, coronary artery bypass grafting, acute myocardial infarction, congestive heart failure, or acute coronary syndrome. Stroke was defined as self-reported doctor-diagnosed stroke, including cerebral hemorrhage and cerebral infarction.

#### 2.2.3 Exposure indices

We examined eight exposure measures, all calculable in both cohorts. BMI was calculated as weight (kg) divided by height squared (m^2^). CVAI was calculated using sex-specific equations: for men, CVAI = −267.93 + 0.68 × age + 0.03 × BMI + 4.00 × WC (cm) + 22.00 × log_10_ TG (mmol/L) − 16.32 × HDL-C (mmol/L); for women, CVAI = −187.32 + 1.71 × age + 4.23 × BMI + 1.12 × WC + 39.76 × log_10_ TG − 11.66 × HDL-C. LAP was calculated as [WC (cm) − 65] × TG (mmol/L) for men and (WC − 58) × TG for women. TyG was calculated as ln [TG (mg/dL) × FPG (mg/dL)/2], and AIP was calculated as log_10_ [TG (mmol/L)/HDL-C (mmol/L)]. hs-CRP was analyzed as a benchmark, and the hs-CRP/HDL-C ratio was calculated by dividing hs-CRP (mg/L) by HDL-C (mg/dL). CTI was calculated as 0.412 × ln [hs-CRP (mg/L)] + TyG.

### 2.3 Statistical analysis

Data processing and statistical analyses were performed using Python 3.9 in Jupyter Notebook (version 7.0.8). Missing data were handled using multiple imputation by chained equations (MICE), generating 10 imputed datasets separately for each cohort. Baseline characteristics were described by CVD status. Continuous variables are presented as mean ± standard deviation, and categorical variables as counts and percentages. Descriptive results were averaged across the 10 imputed datasets. Group differences were assessed using Welch's *t*-test for continuous variables and the chi-square test for categorical variables. A *P* value < 0.05 was considered statistically significant.

For each cohort, Cox proportional hazards models were fitted separately in the 10 imputed datasets to examine the association between each exposure index and incident CVD. Estimates were pooled using Rubin's rules. Model 1 included only the exposure index. Model 2 additionally adjusted for gender, age, WC, SBP, FPG, smoking, weight, drinking, education level, hypertension, and diabetes, excluding variables that were components of the index. Model 3 used a reduced adjustment set, including gender, age, smoking, drinking, and education level, with the same component-exclusion rule. Details for each index–model combination are provided in Supplementary Table S1. Multicollinearity among covariates in Model 2 was assessed using the variance inflation factors (VIF) values, and the proportional hazards assumption was assessed by Schoenfeld residual-based tests. Cohort-interaction analyses were used to examine whether the association between each index and CVD differed between the two cohorts. Quartile-based analyses were conducted to assess dose-response relationships and restricted cubic spline (RCS) analyses to explore potential nonlinear associations. Prediction analyses were performed using a common clinical base model that included age, gender, SBP, TC, HDL-C, smoking, diabetes, and hypertension. Each index was added separately to this base model. Prediction performance was evaluated using the C-index, AUC, and Brier score, with bootstrap optimism correction applied to these metrics. Decision curve analysis (DCA) was used to assess clinical utility by estimating net benefit across threshold probabilities from 1% to 20%. Finally, subgroup analyses were performed by gender, age (<60 vs. ≥60 years), diabetes and hypertension status to assess whether the associations between each index and CVD varied across subgroups.

## 3 Results

### 3.1 Population characteristics

Baseline characteristics of the two cohorts are presented in Table 1. A total of 7,170 participants were included from two cohorts. During a mean follow-up of 40.79 months in CHARLS, 343 participants experienced a CVD event. In SHDS, 148 CVD events occurred over a mean follow-up of 41.28 months. The event rates were 6.4% for CVD, 5.5% for CHD, and 1.0% for stroke in CHARLS, versus 8.2% for CVD, 2.9% for CHD, and 5.3% for stroke in SHDS. In both cohorts, participants with CVD were older, had higher body weight and larger waist circumferences, and were more likely to be female than participants without CVD. They also had higher lipid levels and a greater prevalence of hypertension and diabetes. With the exception of hs-CRP in both cohorts, and BMI, LAP, AIP, and hs-CRP/HDL-C in SHDS, all exposure indices were significantly higher in the CVD group (all *P* < 0.05).

**Table 1:** Baseline characteristics of the two independent cohorts stratified by CVD incidence.

Variables	CHARLS			SHDS		
	No CVD	CVD	*P*-value	No CVD	CVD	*P*-value
	(*n* = 5,023)	(*n* = 343)		(*n* = 1,656)	(*n* = 148)	
Follow-up time, (month)	41.18 ± 16.58	34.95 ± 11.93	<0.001	42.94 ± 11.20	22.74 ± 14.31	<0.001
Age, (years)	57.95 ± 8.90	60.09 ± 8.98	<0.001	62.42 ± 11.27	69.73 ± 8.58	<0.001
Gender			0.041			0.572
Female, *n* (%)	2,620 (52.15%)	199 (58.02%)		941 (56.82%)	80 (54.05%)	
Male, *n* (%)	2,403 (47.85%)	144 (41.98%)		715 (43.18%)	68 (45.95%)	
Height, (cm)	158.76 ± 8.66	158.15 ± 8.84	0.197	160.03 ± 8.47	158.56 ± 8.50	0.046
Weight, (kg)	60.01 ± 12.14	61.11 ± 12.92	0.128	62.38 ± 10.50	62.09 ± 10.41	0.748
WC, (cm)	85.71 ± 10.19	87.62 ± 10.29	<0.001	83.77 ± 9.86	85.32 ± 10.57	0.087
SBP, (mmHg)	130.05 ± 20.52	135.66 ± 22.55	<0.001	130.85 ± 20.41	139.47 ± 20.21	<0.001
DBP, (mmHg)	75.88 ± 11.86	77.93 ± 12.65	0.003	80.72 ± 10.71	82.14 ± 10.46	0.118
Education level						
Primary school or below, *n* (%)	3,180 (63.31%)	223 (65.01%)	0.564	1,113 (67.19%)	114 (76.82%)	0.018
Junior or senior high school, *n* (%)	1,636 (32.56%)	99 (28.86%)	0.175	323 (19.49%)	24 (16.42%)	0.397
College or above, *n* (%)	207 (4.13%)	21 (6.12%)	0.101	221 (13.32%)	10 (6.76%)	0.03
Drinking habit			0.019			0.432
Never drink, *n* (%)	4,563 (90.84%)	299 (87.14%)		1,433 (86.52%)	132 (89.19%)	
Currently drinking, *n* (%)	460 (9.16%)	44 (12.86%)		223 (13.48%)	16 (10.81%)	
Smoking habit			0.964			0.883
Never smoke, *n* (%)	3,075 (61.21%)	211 (61.49%)		1,238 (74.76%)	112 (75.68%)	
Currently smoking, *n* (%)	1,948 (38.79%)	132 (38.51%)		418 (25.24%)	36 (24.32%)	
Diabetes, *n* (%)	241 (4.80%)	23 (6.73%)	0.144	245 (14.79%)	41 (27.70%)	<0.001
Hypertension, *n* (%)	1,052 (20.94%)	126 (36.85%)	<0.001	820 (49.52%)	103 (69.59%)	<0.001
FPG, (mmol/L)	6.1 ± 1.9	6.4 ± 2.6	0.061	5.47 ± 1.54	5.98 ± 2.27	0.008
TC, (mmol/L)	5.00 ± 0.98	5.07 ± 1.03	0.264	5.22 ± 1.12	5.45 ± 1.40	0.053
TG, (mmol/L)	1.50 ± 1.12	1.65 ± 1.12	0.018	1.82 ± 1.03	2.09 ± 2.14	0.135
LDL-C, (mmol/L)	3.03 ± 0.89	3.06 ± 0.95	0.584	3.66 ± 0.97	3.83 ± 1.15	0.091
HDL-C, (mmol/L)	1.32 ± 0.39	1.30 ± 0.42	0.309	1.33 ± 0.26	1.33 ± 0.27	0.8
hs-CRP, (mg/L)	2.40 ± 5.99	3.18 ± 8.06	0.079	2.49 ± 5.35	3.06 ± 4.79	0.173
BMI	23.82 ± 4.53	24.43 ± 4.84	0.022	24.32 ± 3.41	24.72 ± 3.80	0.222
CVAI	101.43 ± 45.53	114.39 ± 45.59	<0.001	112.51 ± 43.56	126.79 ± 45.94	<0.001
LAP	39.37 ± 40.43	46.16 ± 40.87	0.003	43.60 ± 36.57	54.97 ± 80.31	0.09
TyG	8.69 ± 0.66	8.81 ± 0.72	0.003	8.83 ± 0.58	8.96 ± 0.71	0.03
AIP	0.00 ± 0.33	0.05 ± 0.35	0.008	0.09 ± 0.24	0.12 ± 0.28	0.246
hs-CRP/HDL-C	0.05 ± 0.14	0.07 ± 0.16	0.039	0.05 ± 0.10	0.06 ± 0.11	0.144
CTI	8.74 ± 0.83	8.95 ± 0.94	<0.001	8.92 ± 0.82	9.17 ± 0.91	0.002

CHARLS, the Chinese population: the China Health and Retirement Longitudinal Study; SHDS, the Shanghai Diabetes Study; CVD, cardiovascular disease; WC, waist circumference; SBP, systolic blood pressure; DBP, diastolic blood pressure; FPG, fasting plasma glucose; TC, total cholesterol; TG, triglyceride; LDL-C, low-density lipoprotein cholesterol; HDL-C, high-density lipoprotein cholesterol; hs-CRP, high-sensitivity C-reactive protein; BMI, body mass index; CVAI, the Chinese visceral adiposity index; LAP, the lipid accumulation product; TyG, the triglyceride-glucose index; AIP, the atherogenic index of plasma; CTI, the CRP-TyG index. A *P* value < 0.05 was considered statistically significant in this study.

### 3.2 Association between incident CVD and exposure indices

Proportional hazards diagnostics supported the use of Cox models, with only limited index-level signals in CHARLS; targeted time-interaction sensitivity analyses for these signals did not show statistically significant time-varying effects (Supplementary Tables S2, S3), although the estimated associations tended to attenuate over follow-up. Table 2 presents the Cox model results for each index-CVD association. We assessed the association of each index with incident CVD outcomes using two primary Cox regression models: Model 1 (unadjusted) and Model 2 (multivariable adjusted, Model 2 VIF results are in Supplementary Figures S1, S2), with an additional reduced-adjustment Model 3 included as a sensitivity analysis.

**Table 2:** Associations of cardiometabolic and inflammatory indices with incident CVD in two cohorts.

Index	Model 1		Model 2		Model 3	
	HR per SD (95%CI)	*P*	HR per SD (95%CI)	*P*	HR per SD (95%CI)	*P*
**CHARLS**						
BMI	1.122 (0.995, 1.267)	0.061	1.035 (0.887, 1.207)	0.666	1.148 (1.015, 1.298)	0.028
CVAI	1.288 (1.160, 1.431)	<0.001	1.221 (1.070, 1.392)	0.003	1.282 (1.155, 1.424)	<0.001
LAP	1.131 (1.043, 1.227)	0.003	1.073 (0.973, 1.184)	0.158	1.144 (1.055, 1.241)	0.001
TyG	1.183 (1.071, 1.307)	<0.001	1.108 (0.994, 1.235)	0.065	1.182 (1.068, 1.308)	0.001
AIP	1.161 (1.049, 1.286)	0.004	1.080 (0.964, 1.210)	0.182	1.172 (1.057, 1.299)	0.003
hs-CRP	1.124 (1.042, 1.213)	0.003	1.086 (1.002, 1.176)	0.044	1.108 (1.025, 1.197)	0.009
hs-CRP/HDL-C	1.127 (1.046, 1.213)	0.002	1.098 (1.009, 1.194)	0.031	1.119 (1.036, 1.208)	0.004
CTI	1.282 (1.160, 1.417)	<0.001	1.184 (1.059, 1.322)	0.003	1.264 (1.142, 1.400)	<0.001
**SHDS**						
BMI	1.127 (0.962, 1.321)	0.140	1.112 (0.844, 1.467)	0.451	1.142 (0.978, 1.334)	0.094
CVAI	1.403 (1.190, 1.656)	<0.001	1.330 (1.067, 1.656)	0.011	1.369 (1.158, 1.619)	<0.001
LAP	1.207 (1.091, 1.334)	<0.001	1.146 (1.011, 1.300)	0.033	1.223 (1.100, 1.359)	<0.001
TyG	1.190 (1.016, 1.395)	0.032	1.085 (0.905, 1.302)	0.379	1.235 (1.051, 1.452)	0.010
AIP	1.043 (0.885, 1.228)	0.616	1.020 (0.852, 1.221)	0.832	1.130 (0.954, 1.337)	0.157
hs-CRP	1.081 (0.967, 1.207)	0.170	1.034 (0.904, 1.182)	0.628	1.045 (0.928, 1.176)	0.471
hs-CRP/HDL-C	1.090 (0.980, 1.213)	0.112	1.050 (0.927, 1.189)	0.440	1.058 (0.948, 1.180)	0.318
CTI	1.298 (1.108, 1.522)	0.001	1.135 (0.944, 1.363)	0.177	1.270 (1.077, 1.496)	0.004

CHARLS, the Chinese population: the China Health and Retirement Longitudinal Study; SHDS, the Shanghai Diabetes Study; CVD, cardiovascular disease; HR, hazard ratio; SD, standard deviation; BMI, body mass index; CVAI, the Chinese visceral adiposity index; LAP, the lipid accumulation product; TyG, the triglyceride-glucose index; AIP, the atherogenic index of plasma; hs-CRP, high-sensitivity C-reactive protein; HDL-C, high-density lipoprotein cholesterol; CTI, the CRP-TyG index. A *P* value < 0.05 was considered statistically significant in this study.

In CHARLS, most cardiometabolic and inflammatory indices were associated with CVD in Model 1 and Model 3. In Model 2, the associations for BMI, LAP, TyG, and AIP were substantially attenuated and were no longer statistically significant, whereas CVAI, CTI, hs-CRP, and the hs-CRP/HDL-C ratio remained independently associated with incident CVD. For CTI, hs-CRP, and the hs-CRP/HDL-C ratio, the HRs per SD increment in Model 2 were 1.184 (95% CI: 1.059–1.322, *P* = 0.003), 1.086 (95% CI: 1.002–1.176, *P* = 0.044), and 1.098 (95% CI: 1.009–1.194, *P* = 0.031), respectively. The SHDS showed a different pattern. CVAI and LAP remained statistically significant across all three models.

Across the two cohorts, CVAI was the only index that remained consistently associated with incident CVD in all three adjustment models. In CHARLS, the HR per SD increment for CVAI was 1.288 (95% CI: 1.160–1.431, *P* < 0.001) in Model 1, 1.221 (95% CI: 1.070–1.392, *P* = 0.003) in Model 2, and 1.282 (95% CI: 1.155–1.424, *P* < 0.001) in Model 3. In SHDS, the corresponding HRs were 1.403 (95% CI: 1.190–1.656, *P* < 0.001), 1.330 (95% CI: 1.067–1.656, *P* = 0.011), and 1.369 (95% CI: 1.158–1.619, *P* < 0.001).

### 3.3 Cross-cohort consistency of index–outcome associations

We further assessed whether the associations between each index and CVD outcomes differed between CHARLS and SHDS by fitting cohort-by-index interaction terms across Models 1 to Model 3. As shown in Supplementary Table S4, no statistically significant cohort-by-index interactions were observed for CVD across the eight measures in any of the three models (all *P* values for interaction >0.05). The ratios of HRs ranged from 0.918 to 1.114, and all 95% CIs included 1, indicating that the associations were consistent in direction between the two cohorts.

### 3.4 Quartile-based risk estimates and restricted cubic spline analyses

The results of the quartile-based and RCS analyses for incident CVD across each index in both cohorts are presented in Supplementary Figures S3–S6. In CHARLS, most indices showed significant increasing trends in CVD risk across quartiles in Model 1. After adjustment in Model 2, these associations were attenuated, but CVAI, LAP, hs-CRP, hs-CRP/HDL-C, and CTI remained significant. In Model 3, most indices showed significant increasing trends in CVD risk across quartiles, while TyG showed a borderline trend (*P* for trend = 0.052). In SHDS, CVAI, hs-CRP, hs-CRP/HDL-C, and CTI were significantly associated with CVD in Model 1. After adjustment in Model 2, CVAI remained significantly associated with CVD (HR = 1.76, 95% CI: 1.10–2.84; *P* for trend = 0.019). In Model 3, CVAI, hs-CRP, CTI, and TyG showed increasing trends in CVD risk across quartiles.

### 3.5 Predictive performance and incremental value of exposure indices

**Figure 2:**
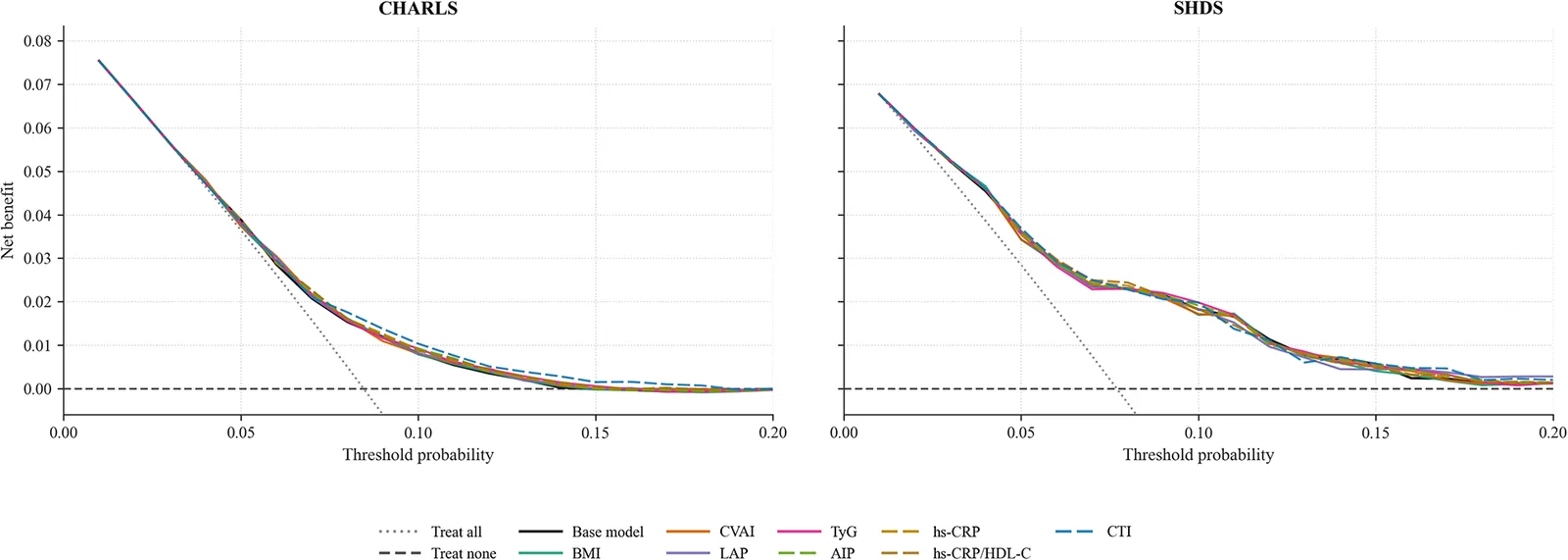
Decision curve analysis for CVD prediction in two cohorts. Decision curves analysis compared the net benefit of a common clinical base model, the base model plus each exposure index, and the default strategies of treating all or no participants across threshold probabilities for incident CVD in CHARLS and SHDS. The base model included age, gender, systolic blood pressure, total cholesterol, HDL-C, smoking status, diabetes, and hypertension. CHARLS, the Chinese population, the China Health and Retirement Longitudinal Study; SHDS, the Shanghai Diabetes Study; CVD, cardiovascular diseases; BMI, body mass index; CVAI, the Chinese visceral adiposity index; LAP, the lipid accumulation product; TyG, the triglyceride-glucose index; AIP, the atherogenic index of plasma; hs-CRP, high-sensitivity C-reactive protein; HDL-C, high-density lipoprotein cholesterol; CTI, the hs-CRP-TyG index.

Predictive performance was evaluated using a common clinical base model, and each index was added separately to assess incremental value. Results are presented in Table 3. After bootstrap optimism correction, the base models showed moderate discrimination, with corrected C-index values of 0.620 for CVD in CHARLS, and 0.694 in SHDS. Adding individual indices produced modest changes in corrected discrimination and calibration. For CVD prediction, the largest corrected improvement in CHARLS was observed after adding CTI, with the corrected C-index increasing from 0.620 to 0.630 and corrected AUC from 0.628 to 0.636, while the Brier score changed minimally. In SHDS, none of the indices materially improved corrected CVD prediction, with corrected C-index values remaining essentially unchanged or slightly lower after index addition. DCA for CVD showed that the net benefit curves for the index-extended models were largely similar to those of the common clinical base model in both cohorts (Figure 2).

**Table 3:** Predictive performance and incremental predictive value of adding cardiometabolic and inflammatory indices to a common clinical base model for CVD prediction in two cohorts.

Model	Optimism-corrected C-index	Optimism-corrected horizon-specific AUC	Optimism-corrected Brier score	Apparent C-index	Apparent horizon-specific AUC	Apparent Brier score
**CHARLS**						
Base model	0.620	0.628	0.077	0.631	0.639	0.077
BMI	0.618	0.625	0.077	0.631	0.638	0.077
CVAI	0.623	0.628	0.077	0.636	0.641	0.076
LAP	0.622	0.629	0.077	0.634	0.641	0.076
TyG	0.623	0.629	0.077	0.635	0.641	0.076
AIP	0.625	0.631	0.077	0.637	0.643	0.076
hs-CRP	0.624	0.633	0.077	0.637	0.646	0.076
hs-CRP/HDL-C	0.625	0.633	0.077	0.638	0.646	0.076
CTI	0.630	0.636	0.076	0.641	0.648	0.076
**SHDS**						
Base model	0.694	0.699	0.069	0.709	0.715	0.068
BMI	0.691	0.696	0.069	0.709	0.715	0.068
CVAI	0.692	0.697	0.069	0.709	0.715	0.068
LAP	0.691	0.697	0.068	0.708	0.714	0.067
TyG	0.693	0.699	0.069	0.709	0.716	0.068
AIP	0.692	0.698	0.069	0.709	0.715	0.068
hs-CRP	0.693	0.699	0.069	0.709	0.716	0.068
hs-CRP/HDL-C	0.693	0.699	0.069	0.710	0.717	0.068
CTI	0.694	0.700	0.069	0.710	0.718	0.068

The common clinical base model included age, gender, systolic blood pressure, total cholesterol, HDL-C, smoking, diabetes, and hypertension. Each exposure index was added separately to this base model. Predicted risks were evaluated at the median follow-up time, corresponding to 48 months in CHARLS and 35 months in SHDS. Optimism-corrected C-index, horizon-specific AUC, and Brier score were estimated using bootstrap optimism correction with 200 resamples within each cohort and imputed dataset. Apparent estimates refer to model performance before optimism correction. Higher C-index and AUC values indicate better discrimination, whereas lower Brier scores indicate better overall prediction accuracy. Estimates were summarized across multiply imputed datasets. CHARLS, the Chinese population: the China Health and Retirement Longitudinal Study; SHDS, the Shanghai Diabetes Study; CVD, cardiovascular disease; CHD, coronary heart disease; BMI, body mass index; CVAI, the Chinese visceral adiposity index; LAP, the lipid accumulation product; TyG, the triglyceride-glucose index; AIP, the atherogenic index of plasma; hs-CRP, high-sensitivity C-reactive protein; HDL-C, high-density lipoprotein cholesterol; CTI, the CRP-TyG index.

### 3.6 Stratified analyses

In stratified analyses of incident CVD, the leading index varied by cohort and subgroup (Supplementary Figures S7, S8). In CHARLS, CVAI had the largest significant Model 3 estimate among women, participants aged <60 years, and those without diabetes or hypertension, with HRs ranging from 1.264 to 1.296 per 1-SD increase. CTI showed larger estimates in men and in participants aged ≥60 years (Model 3 HRs: 1.308 and 1.315, respectively), and remained significant across all three models in women, men, older participants, and those without diabetes or hypertension. In SHDS, CVAI was the most prominent among women, participants aged <60 years, and those with or without diabetes, with Model 3 HRs ranging from 1.249 to 1.825. LAP yielded the largest estimates in men and hypertensive participants, whereas CTI was the highest among non-hypertensive participants. Associations that persisted across Models 1–3 were mainly seen for CVAI in women and participants with diabetes, and for LAP in participants with diabetes or hypertension. Most interaction tests were not significant; the nominally significant findings were limited to sex interactions for hs-CRP and hs-CRP/HDL-C in CHARLS, and age or sex interactions for BMI and CVAI in SHDS.

## 4 Discussion

In This study, we used two Chinese population-based cohorts, CHARLS and SHDS, to compare cardiometabolic and inflammation-related indices in relation to incident CVD. Among the indices examined, CVAI was the only index that maintained a stable and consistent association with CVD risk in both cohorts. Most indices showed positive dose-response associations with CVD risk, though estimates were attenuated after multivariable adjustment. When indices were added to a clinical base model, the addition resulted in modest improvements in predictive performance.

Atherosclerosis is the fundamental pathological substrate for most cardiovascular diseases (2), and it is shaped by several related processes, including insulin resistance, dyslipidemia, chronic low-grade inflammation, and aging (9, 22–24). This provides a rationale for evaluating composite indices that combine anthropometric, metabolic, lipid, and inflammatory information rather than relying only on single markers. In the present study, CVAI showed the most consistent association with CVD risk in both CHARLS and SHDS, whereas LAP remained significant across all models only in SHDS. Both indices reflect abdominal adiposity and lipid-related metabolic disturbance, but they differ in construction. CVAI incorporates age, BMI, WC, TG, and HDL-C, while LAP is based on gender, WC, and TG. This distinction may matter in Chinese population, LAP and VAI were originally developed in Western populations (16, 25), whereas CVAI was adapted from VAI to better reflect body composition and metabolic risk in Chinese adults (15). Previous studies have reported that, at the same BMI level, Chinese adults tend to have higher body fat percentage and greater visceral fat accumulation than Caucasian adults (26–28). By combining age and BMI together with WC and lipid measures, CVAI may better capture differences in metabolic risk that are not reflected by BMI or WC alone. This interpretation is consistent with a recent CHARLS-based study, in which cumulative CVAI demonstrated better CVD predictive performance compared with BMI, WC, and VAI (AUC: 59.83% vs. 55.06%, 55.65%, and 55.08%, respectively), and each 1-SD increase in cumulative CVAI corresponding to a 20% higher CVD risk after full adjustment (HR = 1.20, 95% CI: 1.06–1.37) (29).

Nevertheless, even for CVAI, the most consistently associated index in our study, the effect estimates were attenuated after adjustment for conventional risk factors in Cox models, suggesting that part of its predictive information overlaps with established cardiometabolic risk factors. Similar attenuation has been reported in other cohorts. In a UK Biobank study of nine obesity-related indices and incident CVD, the associations became weaker after sequential adjustment for hypertension, diabetes, and lifestyle factors (30). A multi-cohort analysis using NHANES, the UK Biobank, and the Shanghai Pudong cohort also found that inflammatory markers, including hs-CRP, partly explained the associations between TyG-related indices and CVD among individuals with hypertension (31). In our data, one SD of CVAI corresponded to approximately 45.6 units in CHARLS and 43.9 units in SHDS. Moving from the median CVAI level to about one SD above the median was accompanied by increases in WC of approximately 8.0 cm and 9.1 cm, TG of approximately 0.57 and 0.35 mmol/L, and BMI of approximately 2.4 and 2.8 kg/m^2^, respectively. A 1-SD increase in CVAI therefore represents a visible shift toward greater central adiposity and a less favorable lipid-metabolic profile, rather than a small isolated change in a single biomarker. These indices may assist in identifying individuals at elevated cardiometabolic risk. However, they should be used alongside careful management of established cardiovascular risk factors.

Although several indices were independently associated with incident CVD, their addition to the common clinical base model produced modest improvements in predictive performance. After bootstrap optimism correction, the AUC in CHARLS was 0.628 for the base model, remained 0.628 after adding CVAI, and increased to 0.636 after adding CTI. In SHDS, the base-model AUC was 0.699, and no added index increased it beyond 0.700. DCA likewise showed only small changes in net benefit. Across the prespecified risk thresholds of 5%–15%, the absolute change in net benefit did not exceed 0.0022, and no index showed a consistent advantage over the clinical base model in both cohorts. These findings are consistent with a pooled analysis of 58 prospective cohorts, in which BMI, WC and waist-to-hip ratio remained associated with incident CVD but added little predictive or reclassification value beyond systolic blood pressure, diabetes, and lipid variables (32). Similarly, WC did not substantively improve fatal or non-fatal CVD prediction when added to the Framingham Risk Score or a population-specific clinical model in a large longitudinal cohort (33). Taken together, composite cardiometabolic and inflammatory indices may remain relevant to CVD risk, but they add limited predictive information when conventional clinical models already include their related cardiometabolic factors.

This study has several limitations. First, the estimates may have been affected by residual confounding, measurement error, and selection bias. Data on diet, physical activity, socioeconomic factors, detailed medication use, and lipid-lowering or antihypertensive treatment were unavailable. Baseline CVD history and incident CVD events were based on participant self-report without verification against medical records, which may have introduced outcome misclassification (34, 35). In CHARLS, participants with missing biomarker data were also excluded from the complete-case analysis; selection bias is possible if these participants differed systematically from those retained. Future studies should validate self-reported endpoint events using medical records, prescription databases, or other data sources where feasible. In addition, CHARLS analyses did not incorporate sampling weights, clustering, or stratification; therefore, these findings should be interpreted as results from an unweighted analytic cohort rather than as nationally representative estimates. Second, follow-up was relatively short, with outcomes assessed over approximately 3 to 4 years. The results should therefore be interpreted as evidence regarding short- to mid-term CVD risk prediction and prognostic assessment. We harmonized the follow-up window across CHARLS and SHDS to improve cohort comparability, but differences in geographic coverage, urban-rural composition, baseline age, cardiometabolic risk profile, follow-up duration, and outcome ascertainment may still have affected cohort-specific estimates and statistical power. Finally, our results should be considered in the context of Chinese population. Compared with western populations, Chinese adults may develop visceral fat accumulation and insulin resistance at relatively lower BMI levels. CVAI was developed in Chinese populations to better capture metabolic risk associated with visceral fat, and its consistent performance across the two cohorts supports its relevance in this setting. Future research should validate these findings in more diverse populations and with longer follow-up periods.

## 5 Conclusion

In two Chinese cohorts from different periods and settings, CVAI showed the most consistent association with short- to mid-term incident CVD. The attenuation of estimates after multivariable adjustment, together with limited improvements in prediction metrics and decision curve net benefit, suggests that these indices capture relevant cardiometabolic burden but add little information once conventional risk factors are included. Longer follow-up studies with verified outcomes are needed to determine whether these findings extend to long-term prediction and to more diverse populations.

## Data Availability

The datasets presented in this study can be found in online repositories. The names of the repository/repositories and accession number(s) can be found below: The CHARLS data that support the findings of this study is publicly available (https://charls.charlsdata.com/). The SHDS dataset used during this study is available from the corresponding authors on reasonable request.
