# Supplementary material for: Comparative associations and incremental predictive value of cardiometabolic and inflammatory indices for short- to mid-term cardiovascular disease risk in two Chinese cohorts
Source: Front Cardiovasc Med. 2026 Aug 6;13:1896146. doi: 10.3389/fcvm.2026.1896146 (PMC13489905; doi:10.3389/fcvm.2026.1896146)
Supplement: Supplementary file 1 [file Datasheet1.docx]

[Supplementary Tables	2](#_Toc235615540)

[Table S1 Components of Cox models for the cardiometabolic and inflammatory indices used in this study	2](#_Toc235615541)

[Table S2. Proportional hazards assumption tests of Cox models for incident CVD	3](#_Toc235615542)

[Table S3 Time-interaction analyses for models showing potential proportional hazards assumption violations	3](#_Toc235615543)

[Table S4. Consistency of associations between cardiometabolic and inflammatory indices and CVD across two cohorts.	4](#_Toc235615544)

[Supplementary Figures	5](#_Toc235615545)

[Figure S1 VIF of the exposure index in Model 2 across cohorts and indices	5](#_Toc235615546)

[Figure S2 Maximum VIF of Model 2 predictors across cohorts and indices	5](#_Toc235615547)

[Figure S3. Quartile-based hazard ratios of exposure indices for incident CVD in CHARLS.	6](#_Toc235615548)

[Figure S4. Quartile-based hazard ratios of exposure indices for incident CVD in SHDS.	6](#_Toc235615549)

[Figure S5. Restricted cubic spline analysis of exposure indices and incident CVD in CHARLS.	7](#_Toc235615550)

[Figure S6. Restricted cubic spline analysis of exposure indices and incident CVD in SHDS.	7](#_Toc235615551)

[Figure S7. Subgroup associations of exposure indices with incident CVD in the CHARLS cohort.	8](#_Toc235615552)

[Figure S8. Subgroup associations of exposure indices with incident CVD in the SHDS cohort.	9](#_Toc235615553)

[Supplementary Section: Assessment of potential selection bias	9](#_Toc235615554)

# **Supplementary Tables**

## **Table S1 Components of Cox models for the cardiometabolic and inflammatory indices used in this study**

| **Index** | **Model** | **Model Components** |
| --- | --- | --- |
| BMI | 1 | BMI |
|  | 2 | BMI, Gender, Age, Waist Circumference, SBP, FPG, Smoking, Drinking, Education Level, Hypertension, Diabetes |
|  | 3 | BMI, Gender, Age, Smoking, Drinking, Education Level |
| CVAI | 1 | CVAI |
|  | 2 | CVAI, SBP, FPG, Smoking, Weight, Drinking, Education Level, Hypertension, Diabetes |
|  | 3 | CVAI, Smoking, Drinking, Education Level |
| LAP | 1 | LAP |
|  | 2 | LAP, Age, SBP, FPG, Smoking, Weight, Drinking, Education Level, Hypertension, Diabetes |
|  | 3 | LAP, Age, Smoking, Drinking, Education Level |
| TyG | 1 | TyG |
|  | 2 | TyG, Gender, Age, Waist Circumference, SBP, Smoking, Weight, Drinking, Education Level, Hypertension, Diabetes |
|  | 3 | TyG, Gender, Age, Smoking, Drinking, Education Level |
| AIP | 1 | AIP |
|  | 2 | AIP, Gender, Age, Waist Circumference, SBP, FPG, Smoking, Weight, Drinking, Education Level, Hypertension, Diabetes |
|  | 3 | AIP, Gender, Age, Smoking, Drinking, Education Level |
| hs-CRP | 1 | hs-CRP |
|  | 2 | hs-CRP, Gender, Age, Waist Circumference, SBP, FPG, Smoking, Weight, Drinking, Education Level, Hypertension, Diabetes |
|  | 3 | hs-CRP, Gender, Age, Smoking, Drinking, Education Level |
| hs-CRP /HDL | 1 | hs-CRP/HDL |
|  | 2 | hs-CRP/HDL, Gender, Age, Waist Circumference, SBP, FPG, Smoking, Weight, Drinking, Education Level, Hypertension, Diabetes |
|  | 3 | hs-CRP/HDL, Gender, Age, Smoking, Drinking, Education Level |
| CTI | 1 | CTI |
|  | 2 | CTI, Gender, Age, Waist Circumference, SBP, Smoking, Weight, Drinking, Education Level, Hypertension, Diabetes |
|  | 3 | CTI, Gender, Age, Smoking, Drinking, Education Level |

Note: Model 1 was unadjusted. Model 2 was the main multivariable model excluding direct components of the corresponding exposure index. Model 3 represents a reduced non-overlapping sensitivity model.

## **Table S2. Proportional hazards assumption tests of Cox models for incident CVD**

| **Index** | **Model1** | | **Model2** | | **Model3** | |
| --- | --- | --- | --- | --- | --- | --- |
|  | **Global P** | **Median Index P** | **Global P** | **Median Index P** | **Global P** | **Median Index P** |
| CHARLS | | | | | | |
| BMI | 0.54 | 0.54 | 0.88 | 0.45 | 0.81 | 0.53 |
| CVAI | 0.39 | 0.39 | 0.77 | 0.31 | 0.50 | 0.37 |
| LAP | 0.48 | 0.48 | 0.89 | 0.56 | 0.74 | 0.55 |
| TyG | 0.35 | 0.35 | 0.90 | 0.34 | 0.71 | 0.39 |
| AIP | 0.41 | 0.41 | 0.94 | 0.56 | 0.73 | 0.43 |
| hs-CRP | 0.18 | 0.18 | 0.86 | 0.19 | 0.62 | 0.21 |
| hs-CRP/HDL | 0.20 | 0.20 | 0.88 | 0.24 | 0.66 | 0.24 |
| CTI | 0.06 | 0.06 | 0.59 | 0.05 | 0.38 | 0.07 |
| SHDS | | | | | | |
| BMI | 0.50 | 0.50 | 0.83 | 0.56 | 0.62 | 0.49 |
| CVAI | 0.91 | 0.91 | 0.98 | 0.81 | 0.85 | 0.90 |
| LAP | 0.88 | 0.88 | 0.99 | 0.88 | 0.86 | 0.83 |
| TyG | 0.62 | 0.62 | 0.17 | 0.70 | 0.65 | 0.65 |
| AIP | 0.79 | 0.79 | 0.19 | 0.67 | 0.64 | 0.74 |
| hs-CRP | 0.73 | 0.73 | 0.19 | 0.68 | 0.64 | 0.64 |
| hs-CRP/HDL | 0.68 | 0.68 | 0.19 | 0.64 | 0.64 | 0.62 |
| CTI | 0.71 | 0.71 | 0.15 | 0.68 | 0.66 | 0.81 |

Note: Global P is the median model-level proportional hazards test P value across the 10 imputed datasets. Median Index P is the median Schoenfeld residual-based test P value for the exposure index across the 10 imputed datasets.

## **Table S3 Time-interaction analyses for models showing potential proportional hazards assumption violations**

| **Model** | **Time-interaction P value** | **HR at 24 months** | **HR at 36 months** | **HR at 48 months** |
| --- | --- | --- | --- | --- |
| CHARLS CHD–hs-CRP | 0.067 | 1.125 (1.020–1.240) | 0.945 (0.784–1.139) | 0.835 (0.613–1.137) |
| CHARLS CHD–hs-CRP/HDL-C | 0.097 | 1.140 (1.029–1.262) | 0.984 (0.830–1.168) | 0.887 (0.670–1.175) |
| CHARLS CVD–CTI | 0.078 | 1.282 (1.114–1.476) | 1.150 (1.024–1.291) | 1.064 (0.903–1.254) |

Note: Because three CHARLS index-outcome associations showed index-level Schoenfeld residual signals, we conducted targeted sensitivity analyses by adding index × log(time/24 months) interaction terms. After pooling across imputations, none of the time-interaction terms reached conventional statistical significance (P = 0.067-0.097), although the time-specific HRs suggested attenuation of the associations over follow-up.

## **Table S4. Consistency of associations between cardiometabolic and inflammatory indices and CVD across two cohorts.**

|  | Model 1 | | Model 2 | | Model 3 | |
| --- | --- | --- | --- | --- | --- | --- |
| **Index** | Ratio of HRs (CHARLS/SHDS), 95% CI | P for interaction | Ratio of HRs (CHARLS/SHDS), 95% CI | P for interaction | Ratio of HRs (CHARLS/SHDS), 95% CI | P for interaction |
| AIP | 1.114 (0.919, 1.350) | 0.273 | 1.099 (0.903, 1.337) | 0.347 | 1.090 (0.897, 1.325) | 0.386 |
| BMI | 0.996 (0.816, 1.216) | 0.968 | 1.079 (0.882, 1.320) | 0.462 | 1.049 (0.863, 1.274) | 0.632 |
| hs-CRP | 1.040 (0.909, 1.190) | 0.566 | 1.036 (0.887, 1.210) | 0.655 | 1.042 (0.907, 1.198) | 0.560 |
| hs-CRP/HDL-C | 1.033 (0.907, 1.177) | 0.624 | 1.031 (0.887, 1.200) | 0.690 | 1.043 (0.912, 1.192) | 0.539 |
| CTI | 0.987 (0.819, 1.191) | 0.895 | 1.032 (0.850, 1.252) | 0.751 | 1.001 (0.826, 1.212) | 0.996 |
| CVAI | 0.918 (0.755, 1.116) | 0.390 | 0.946 (0.773, 1.156) | 0.585 | 0.921 (0.757, 1.121) | 0.411 |
| LAP | 0.938 (0.824, 1.067) | 0.329 | 0.949 (0.822, 1.094) | 0.468 | 0.942 (0.825, 1.074) | 0.372 |
| TyG | 0.994 (0.824, 1.199) | 0.947 | 1.029 (0.848, 1.248) | 0.772 | 0.994 (0.822, 1.202) | 0.951 |

Note: The ratio of HRs was calculated as the hazard ratio per 1-SD increase in each index in CHARLS divided by the corresponding hazard ratio in SHDS; indices were standardized separately within each cohort. P for interaction tests whether the index–outcome association differs between cohorts.

# **Supplementary Figures**

## **Figure S1 VIF of the exposure index in Model 2 across cohorts and indices**

Note: The heatmap shows the median VIF of the exposure index itself across 10 imputed datasets for each cohort-index combination. Rows represent cohorts, and columns represent indices. This figure evaluates whether each index was highly collinear with the adjusted covariates in Model 2. VIF values below 5 generally indicate low concern for multicollinearity.

## **Figure S2 Maximum VIF of Model 2 predictors across cohorts and indices**

Note: The heatmap shows the median value across 10 imputed datasets of the maximum variance inflation factor (VIF) among all predictors included in Model 2 for each cohort-index combination. Rows represent cohorts, and columns represent indices. Model 2 included the index of interest and adjusted covariates after excluding direct index components where applicable. VIF values below 5 generally indicate low concern for multicollinearity.

## **Figure S3. Quartile-based hazard ratios of exposure indices for incident CVD in CHARLS.**

Note: Quartile-based Cox models showed HRs for incident CVD across quartiles of each index in CHARLS. Q1 is the reference group. HRs above 1 indicate higher risk compared with Q1. Error bars represent 95% confidence intervals. If the confidence interval does not cross 1, the quartile-specific association is statistically significant. The p for trend evaluates whether risk increases or decreases progressively from Q1 to Q4.

## **Figure S4. Quartile-based hazard ratios of exposure indices for incident CVD in SHDS.**

Note: Quartile-based Cox models showed HRs for incident CVD across quartiles of each index in SHDS. Q1 is the reference group. HRs above 1 indicate higher risk compared with Q1. Error bars represent 95% confidence intervals. If the confidence interval does not cross 1, the quartile-specific association is statistically significant. The p for trend evaluates whether risk increases or decreases progressively from Q1 to Q4.

## **Figure S5. Restricted cubic spline analysis of exposure indices and incident CVD in CHARLS.**

Note: Restricted cubic spline analysis shows the dose-response associations between each index and incident CVD in both cohorts. The x-axis represents the continuous value of each index, and the y-axis represents the hazard ratio (HR) relative to the median value of that index. The horizontal dashed line indicates HR = 1. Curves above 1 suggest higher risk, while curves below 1 suggest lower risk. Shaded areas represent 95% confidence intervals. Model 1, Model 2, and Model 3 are shown separately to compare whether the association remains stable after different levels of adjustment.

## **Figure S6. Restricted cubic spline analysis of exposure indices and incident CVD in SHDS.**

Note: Restricted cubic spline analysis shows the dose-response associations between each index and incident CVD in both cohorts. The x-axis represents the continuous value of each index, and the y-axis represents the hazard ratio (HR) relative to the median value of that index. The horizontal dashed line indicates HR = 1. Curves above 1 suggest higher risk, while curves below 1 suggest lower risk. Shaded areas represent 95% confidence intervals. Model 1, Model 2, and Model 3 are shown separately to compare whether the association remains stable after different levels of adjustment.

## **Figure S7. Subgroup associations of exposure indices with incident CVD in the CHARLS cohort.**

## **Figure S8. Subgroup associations of exposure indices with incident CVD in the SHDS cohort.**

# **Supplementary Section: Assessment of potential selection bias**

In CHARLS, the complete-biomarker restriction was applied before multiple imputation. Participants with missing values in any of fasting plasma glucose, total cholesterol, triglycerides, HDL cholesterol, LDL cholesterol, or hs-CRP were excluded because these observed values were required to calculate the prespecified exposure indices. Multiple imputation by chained equations was subsequently used only for other missing covariates among retained participants. To assess potential selection bias, we compared observed baseline characteristics between participants retained and excluded by this restriction using standardized mean differences (SMDs).

Among 8,088 CHARLS participants eligible for follow-up, 5,366 had complete values for all six required biomarkers and were retained, whereas 2,722 (33.65%) were excluded because at least one required biomarker was missing. Compared with excluded participants, those retained in the analysis were slightly older and shorter, and had higher proportions of women and participants with lower education levels. Differences with an absolute SMD of at least 0.10 were observed for Age, years (SMD=0.11); Height, cm (SMD=0.12); Sex (Female) (SMD=0.12); Education level (Primary school or below) (SMD=0.16). These differences were small to moderate, with absolute SMDs ranging from 0.11 to 0.16. 2,696 excluded participants were missing all six blood biomarkers, suggesting that the missingness was more likely related to incomplete blood testing as a whole rather than isolated missing values for individual biomarkers. CVD event rates were similar between the two groups, at 6.4% and 5.9% respectively, with an SMD of 0.02. Follow-up duration differed, however, at 40.79 versus 36.53 months, with an SMD of 0.23.

Baseline characteristics of CHARLS participants included in and excluded from the analytic cohort.

| **Characteristic** | **Included** | **Excluded** | **SMD** | **P Value** |
| --- | --- | --- | --- | --- |
| Age, years | 58.09 ± 8.92 | 57.04 ± 9.28 | 0.11 | <0.001 |
| Height, cm | 158.59 ± 8.51 | 159.62 ± 8.54 | -0.12 | <0.001 |
| Weight, kg | 59.72 ± 11.45 | 60.05 ± 12.04 | -0.03 | 0.323 |
| Waist circumference, cm | 84.50 ± 12.62 | 84.36 ± 12.29 | 0.01 | 0.692 |
| Systolic blood pressure, mmHg | 130.05 ± 20.65 | 129.02 ± 20.25 | 0.05 | 0.069 |
| Diastolic blood pressure, mmHg | 75.75 ± 11.88 | 75.95 ± 11.84 | -0.02 | 0.54 |
| Sex |  |  |  |  |
| Female | 2817 (52.5%) | 1269 (46.6%) | 0.12 | <0.001 |
| Male | 2547 (47.5%) | 1452 (53.4%) | -0.12 | <0.001 |
| Education level |  |  |  |  |
| Primary school or below | 3397 (63.4%) | 1501 (55.4%) | 0.16 | <0.001 |
| Junior or senior high school | 1732 (32.3%) | 1015 (37.5%) | -0.11 | <0.001 |
| College or above | 228 (4.3%) | 192 (7.1%) | -0.12 | <0.001 |
| Smoking |  |  |  |  |
| Never smoke | 3274 (61.2%) | 1546 (57.7%) | 0.07 | 0.003 |
| Currently smoking | 2076 (38.8%) | 1133 (42.3%) | -0.07 | 0.003 |
| Drinking |  |  |  |  |
| Never drink | 3676 (93.5%) | 1814 (93.6%) | -0.00 | 0.869 |
| Currently drinking | 256 (6.5%) | 124 (6.4%) | 0.00 | 0.869 |
| Hypertension | 1170 (22.0%) | 480 (18.0%) | 0.10 | <0.001 |
| Diabetes | 262 (4.9%) | 108 (4.1%) | 0.04 | 0.076 |
| Follow-up time, months | 40.79 ± 16.39 | 36.53 ± 19.79 | 0.23 | <0.001 |
| Follow-up CVD (0) | 5023 (93.6%) | 2561 (94.1%) | -0.02 | 0.401 |
| Follow-up CVD (1) | 343 (6.4%) | 161 (5.9%) | 0.02 | 0.401 |
| Incident CHD (0) | 5070 (94.5%) | 2592 (95.2%) | -0.03 | 0.159 |
| Follow-up CHD (1) | 296 (5.5%) | 130 (4.8%) | 0.03 | 0.159 |
| Incident stroke (0) | 5310 (99.0%) | 2688 (98.8%) | 0.02 | 0.405 |
| Follow-up stroke (1) | 56 (1.0%) | 34 (1.2%) | -0.02 | 0.405 |

Missing data for variables included in the SHDS analysis

| Variable | Missing, n (%) |
| --- | --- |
| Follow-up time, month | 0 (0.00) |
| Age, years | 0 (0.00) |
| Gender | 0 (0.00) |
| Height | 0 (0.00) |
| Weight | 0 (0.00) |
| WC | 0 (0.00) |
| SBP | 0 (0.00) |
| DBP | 0 (0.00) |
| Education level | 45 (1.69) |
| Drinking | 31 (1.16) |
| Smoking | 35 (1.31) |
| Diabetes | 20 (0.75) |
| Hypertension | 0 (0.00) |
| FPG | 0 (0.00) |
| TC | 0 (0.00) |
| TG | 0 (0.00) |
| LDL-C | 0 (0.00) |
| HDL-C | 0 (0.00) |
| hs-CRP, mg/L | 1 (0.04) |
